# Alternative Therapeutic Approaches in the Management of Gastroparesis: A Systematic Review

**DOI:** 10.3390/diseases14050179

**Published:** 2026-05-20

**Authors:** Desaree Tan, Serena Singh, Usha Krishnan, Vincent Ho

**Affiliations:** 1 Melbourne Medical School, The University of Melbourne, Melbourne, VIC 3010, Australia; desaree.z.tan@gmail.com; 2 Department of Gastroenterology, Wollongong Hospital, Wollongong, NSW 2500, Australia; serena.puransingh@health.nsw.gov.au; 3 Department of Gastroenterology, Sydney Children’s Hospital, Sydney, NSW 2031, Australia; usha.krishnan@health.nsw.gov.au; 4 School of Medicine, Western Sydney University, Sydney, NSW 2000, Australia

**Keywords:** gastroparesis, acupuncture, electroacupuncture, moxibustion, herbal medicines

## Abstract

Background: Gastroparesis is characterised by prolonged gastric emptying in the absence of mechanical gastric obstruction. When symptomatic, gastroparesis significantly impacts quality of life. While current consensus emphasises medical, surgical, or nutritional therapies, data evaluating the efficacy of alternative therapies remains scarce. Here, we review the efficacy of alternative therapies; acupuncture, electroacupuncture, moxibustion, and herbal medicine as management strategies. Methods: A systematic literature review of the literature was performed until February 2025. All papers were published from 2001 to 2024. This search focusses on the efficacy of acupuncture, electroacupuncture, moxibustion, and herbal medicines for management of gastroparesis. A comprehensive search was performed in PubMed, Embase, Medline, Google Scholar, Science Direct, and Web of Science. There were no language restrictions. Study outcomes were compared in a narrative synthesis and quality was assessed using Critical Appraisal Skills Programme (CASP) checklists. Results: We identified 68 studies of acupuncture, electroacupuncture, moxibustion, and herbal medicine as alternate therapies for gastroparesis. The total patient sample size of included studies was 4566, with a mean sample of 70.25 per study. The focus of studies comprising our review was electroacupuncture (11%), moxibustion (11%), acupuncture (29%), and herbal medicine (49%). Control comparisons were made with Mosapride, Metoclopramide, Domperidone, and Itopride. Conclusions: We found that alternative therapies are effective for the management of gastroparesis. However, the review was limited by heterogeneous study designs, incomplete methodological reporting, and publication bias. Future investigations must focus on long-term randomised control trials encompassing large sample sizes.

## 1. Introduction

Gastroparesis (GP) is a condition characterised by prolonged gastric emptying in the absence of a mechanical gastric obstruction [1]. It is usually, but not always, associated with distressing symptoms such as post-prandial fullness, early satiety, nausea, vomiting, and bloating. This disorder and most of its subtypes (idiopathic, diabetic, post-surgery, post-infective) have been discussed in the literature since the mid-1900s [2,3]. Other causes of gastroparesis include adverse effects of medications, neurological diseases, connective tissue diseases, and renal insufficiency [4]. Recently, there has been a renewed interest in improving our understanding of the pathophysiological mechanisms of gastroparesis as well as developing a more tailored therapeutic approach [4,5].

The cost of managing GP in the healthcare system is substantial. This could be attributed to the necessity for hospitalisation and diagnostic tests such as endoscopy and gastric emptying scintigraphy [6]. Hospitalisations for GP more than doubled in the United States between 1995 and 2004, indicating an increasing incidence [7]. Unfortunately, the weak connection between gastric emptying and symptoms makes it difficult to estimate incidence precisely [8]. In one study, GP was associated with the most hospitalisations among upper gastrointestinal disorders [7]. The reasons for the rising incidence of GP-related hospitalisations are unknown. The rising incidence of GP may partly be explained by its association with diabetes. Other possible explanations include changes in clinical definition, severity, and management of GP and improved identification and detection [7].

The pathophysiology of delayed gastric emptying includes autonomic neuropathy, enteric neuropathy, loss of Interstitial cells of Cajal (ICC), the pacemaker cells that begin slow waves in the stomach, as well as both acute and chronic hyperglycaemia [9]. In full-thickness gastric biopsy specimens, which were available for 40 patients (83%) with either diabetic or idiopathic GP, cellular abnormalities of ICC, aberrant immunological infiltrates, and damaged nerve fibres were identified [10]. The effect of acute hyperglycaemia on gastric emptying and its treatment with prokinetic drugs is well understood, but the influence of chronic hyperglycaemia is less apparent [11]. Post-surgical gastroparesis occurs due to loss of antral contractions and pyloric relaxation as a result of vagus nerve injury following upper abdominal surgery [12].

Currently, dietary modifications and nutritional support, optimal glycaemic control, pharmacological agents (prokinetic drugs), endoscopic interventions (endoscopic injection of botulinum toxin), and surgery are all used to treat gastroparesis [1]. Recent guidance from the American Gastroenterological Association focusses on dietary modifications and prokinetic pharmacotherapy as first-line management strategies for GP. Yet, these recommendations reflect the limited availability of evidence, as these are conditional recommendations with low certainty [13]. Key prokinetic medications such as metoclopramide and erythromycin are associated with adverse effects such as extrapyramidal symptoms, tardive dyskinesia, and tachyphylaxis which restrict long term use [13]. Other pharmacological agents including domperidone and tricyclic neuromodulators remain variably accessible and are associated with anticholinergic side effects [13].

Consequently, GP patients experience persistent symptoms despite guideline-directed therapy. Importantly, these guidelines display limited evaluation of alternative therapies. In this review, alternative therapies refer to non-pharmacological interventions not currently incorporated into guideline-directed management of GP. Focus will therefore centre on acupuncture (AC), electroacupuncture (EA), moxibustion (MX), and herbal medicine (HM). These interventions are supported by emerging mechanistic evidence which focusses on modulation of vagal activity, ICC signalling, and gastrointestinal hormonal regulation [14,15,16]. As GP is increasingly recognised as a disorder of complex neuro-enteric dysfunction, therapies focussing on autonomic and pacemaker-cell pathways may offer clinically adjunctive benefit with lesser adverse effects [5]. Therefore, a systematic synthesis of alternative management strategies for the treatment of GP is warranted to address this gap in current guideline-based management and highlight potential clinical efficacy in alternative therapies.

## 2. Materials and Methods

### 2.1. Protocol

This systematic review was carried out in accordance with the recommended reporting guidelines for systematic reviews and meta-analyses (PRISMA). A literature evaluation was completed in February 2025 using PubMed, Embase, Ovid, Google Scholar, ScienceDirect, and Web of Science. Various combinations of keywords were used in the search parameters, including “alternative treatment for gastroparesis,” “acupuncture for management of gastroparesis,” “electroacupuncture for management of gastroparesis,” “moxibustion therapy for management of gastroparesis,” “herbal medicines for the management of gastroparesis,” “pre and probiotic therapy for management of gastroparesis.” Equivalent keywords were also searched for within article titles, abstracts, and keywords. The titles and abstracts of the produced results were used to filter the findings. Duplicate articles were deleted. The research protocol was registered with PROSPERO (Registration ID number CRD420261364653).

### 2.2. Eligibility Criteria

Studies that were eligible for inclusion if they were published between 2001 and 2024, in any geographic location, and written in English. There were no restrictions on sex within cohorts. All studies including case reports and case series that met the objective of the review, management of GP via AC, EA, MX, PPB and HM, were included. To meet inclusion criteria, studies must include adult patients (≥18 years) with GP who have underwent treatment with alternative therapies (AC, EA, MX, PPB and HM) and reported outcomes of GP symptomatic and clinical management.

Studies were excluded if they were published before 2001 or were not peer-reviewed full text articles. Case reports, letters to editor, commentaries, conference abstracts, and grey literature were excluded from this review. Studies which focussed on paediatric patients (≤18 years) will also not be included in this review.

### 2.3. Search Strategy

#### 2.3.1. Ovid and Embase Shared Search Strategy

(exp Gastroparesis/OR gastroparesis.ti,ab. OR gastric stasis.ti,ab. OR delayed gastric emptying.ti,ab.) AND (exp Complementary Therapies/OR exp Acupuncture Therapy/OR exp Electroacupuncture/OR exp Moxibustion/OR exp Homeopathy/OR exp Herbal Medicine/OR exp Prebiotics/OR exp Probiotics/OR exp Fecal Microbiota Transplantation/OR acupuncture.ti,ab. OR moxibustion.ti,ab. OR homeopath*.ti,ab. OR herbal medicine*.ti,ab. OR prebiotic*.ti,ab. OR probiotic*.ti,ab. OR fecal microbiota.ti,ab. OR complementary therap*.ti,ab. OR alternative therap*.ti,ab.) AND yr = “2001–2024”.

#### 2.3.2. PubMed Search Strategy

(“Gastroparesis”[Mesh] OR gastroparesis[tiab] OR “gastric stasis”[tiab] OR “delayed gastric emptying”[tiab]) AND (“Complementary Therapies”[Mesh] OR “Acupuncture Therapy”[Mesh] OR electroacupuncture[Mesh] OR moxibustion[Mesh] OR homeopathy[Mesh] OR “Herbal Medicine”[Mesh] OR prebiotics[Mesh] OR probiotics[Mesh] OR “Fecal Microbiota Transplantation”[Mesh] OR acupuncture[tiab] OR moxibustion[tiab] OR homeopath*[tiab] OR herbal medicine*[tiab] OR prebiotic*[tiab] OR probiotic*[tiab] OR “fecal microbiota”[tiab] OR complementary therap*[tiab] OR alternative therap*[tiab]) AND (“1 January 2001”[PDAT]:“31 December 2024”[PDAT]).

#### 2.3.3. ScienceDirect Search Strategy

(“gastroparesis” OR “gastric stasis” OR “delayed gastric emptying”) AND (“complementary therapies” OR “acupuncture therapy” OR electroacupuncture OR moxibustion OR homeopathy OR “herbal medicine” OR prebiotics OR probiotics OR “fecal microbiota transplantation” OR acupuncture OR complementary therapy OR alternative therapy OR “fecal microbiota”) AND 2001–2024.

#### 2.3.4. Web of Science Search Strategy

TS = (gastroparesis OR “gastric stasis” OR “delayed gastric emptying”) AND TS = (“complementary therapies” OR “acupuncture therapy” OR electroacupuncture OR moxibustion OR homeopathy OR “herbal medicine” OR prebiotic* OR probiotic* OR “fecal microbiota transplantation” OR acupuncture OR complementary therap* OR alternative therap* OR “fecal microbiota”).

Filter: 2001–2024.

### 2.4. Study Selection

The study selection process was conducted by two reviewers (D. Z. L. T & S. S). The screening process was blinded and each reviewer worked independently. With the use of Covidence software, all papers from databases were collated and duplicates were removed. Initial screening of titles and abstracts of retrieved records was performed to check for relevance. Subsequently, full-text articles were assessed against the eligibility criteria. Exclusion and inclusion criteria were defined prior to the creation of search strategies and screening by the review team. Articles not meeting the inclusion criteria were excluded after this stage. During the process, disagreements at both stages were discussed to arrive to a consensus. If an agreement could not be made, a third reviewer made the final decision.

### 2.5. Data Extraction and Quality Assessment

Articles were then analysed for relevant information and data were extracted and were independently keyed into Excel spreadsheets by two reviewers. Extracted outcomes focussed on study characteristics, including number of patients and trial groups, intervention details, duration, and clinical outcomes. Outcomes as mandated by the paper for symptomatic and clinical measures of gastroparesis were reported. Extracted data was then cross-checked between both reviewers. Inconsistencies were mediated by discussion or a third reviewer if required. The investigators did not contact any author for missing or additional data. Therefore, a narrative synthesis of the data was performed. Risk of bias was assessed independently by two reviewers using the Critical Appraisal Skills Programme (CASP) checklist. A total of 65 studies were appraised using and discrepancies were resolved between reviewers through discussion. A third reviewer will be consulted when required. Risk of bias informed later interpretation of study findings, however, did not lead to the exclusion of studies. Meta-analysis was not conducted due to the heterogeneity and broad nature of this study.

## 3. Results

**Figure 1:**
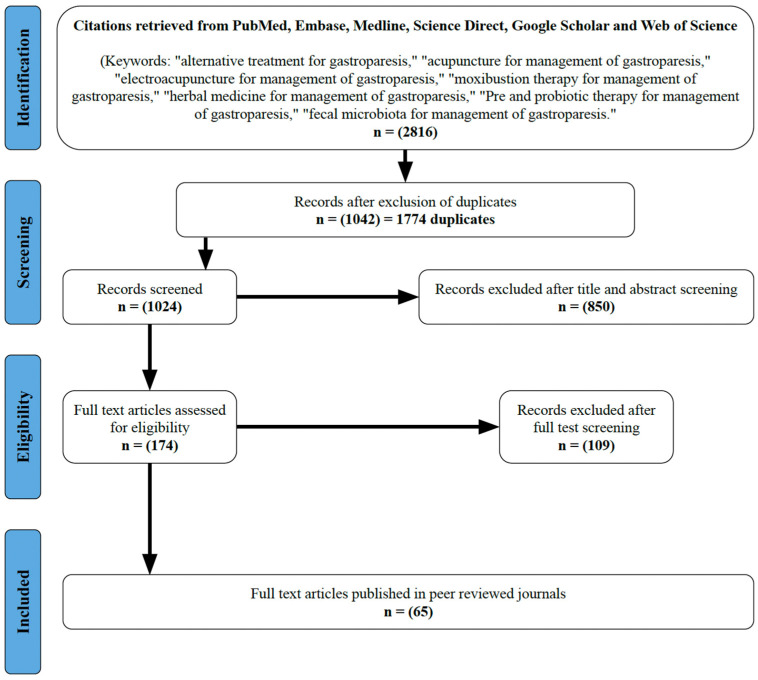
PRISMA flow diagram.

The systematic search identified 2816 articles using PubMed, Embase, Ovid, Google Scholar, Web of Science, and Science Direct. Of these, 1774 were duplicates and were excluded, leaving 1024 relevant articles. Then, 850 additional articles were excluded following a screening of the title and abstract (remaining *n* = 174) and another 106 were excluded following full-text screening (remaining *n* = 65). These final 65 studies [17,18,19,20,21,22,23,24,25,26,27,28,29,30,31,32,33,34,35,36,37,38,39,40,41,42,43,44,45,46,47,48,49,50,51,52,53,54,55,56,57,58,59,60,61,62,63,64,65,66,67,68,69,70,71,72,73,74,75,76,77,78,79,80,81,82] were chosen for the review (Figure 1). All studies reviewed were published between 1 January 2001 and 31 December 2024. The trial’s cumulative patient sample size was 4566, with a mean sample size of 70.25. The distribution of GP treatments among the included studies was EA (11%), MX (11%), AC (29%), and HM (49%). Control comparison groups, which consisted of patients who did not receive the intervention were treated with Mosapride, Metoclopramide, Domperidone, or Itopride. Of 65 studies, one study combined Banxia-Xiexin decoction with moxibustion [26], and one trial used Chinese herb partitioned with moxibustion [25]. Three studies used Mosapride [29,41,54], six studies used Metoclopramide [22,34,37,38,42,71], twenty-five utilised Domperidone [20,30,32,35,39,40,44,45,46,49,50,51,52,53,56,57,58,59,60,61,63,64,67,81,82], and three used Itopride [25,68,70] as baseline management within the control group. The characteristics of the included studies are presented in Table 1.

### 3.1. Therapeutic Response

The response rate of the interventions varied among the studies included in this review. Interventions such as AC, EA, MX, and HM solely or in combination were positive in a few studies. For example, Zhou et al. [18] studied the effect of electroacupuncture at lower *he*-sea point and *he*-sea matching front-*mu* points for the treatment of GP. A total of 63 patients were divided into two groups: a lower *he*-point group (group A) and a *he* matching *mu* point group (group B). Group A received EA at *Zusanli* (ST 36) while group B received EA at *Zusanli* (ST 36) along with *Zhongwan* (CV 12). The wave frequency was kept at 2 Hz for 30 min, once a day, five times a week for three weeks. The main readout of this study was gastroparesis cardinal symptom index (GCSI) score, 180 min gastric residual rate (GRR) and gastric half emptying time (T_1/2_) observed before and after treatment. The conclusion was that the total effective rate of group A was 93.3% which was significantly different (*p* ˂ 0.05) from group B (70.0%). Similarly, Sun and Wang [17] reported a significant therapeutic response by comparing MX, routine AC, and AC with auricular point sticking in 41 postsurgical GP patients. The warming needle MX was effective for postsurgical GP with a therapeutic response of 100% compared to 66.7% and 75.0% in group B and C, who received AC with auricular point sticking and routine AC, respectively. In another study, AC was compared with 5 mg Mosapride citrate for the treatment of diabetic GP [29]. The AC (*Tiaoli Piwei*) technique was applied to *Zhonwen* (CV 12), *Zusanli* (ST 36), *Yinlingquan* (SP 9), *Xuehai* (SP 10), *Sanyinjiao* (SP 6), *Diji* (SP 8) points. The treatment was given six times a week for four weeks. The authors determined that AC was better than Mosapride citrate (control) in treating gastroparesis, i.e., the therapeutic response was 86.7% for the treatment groups as compared to 70.0% for the control group (*p* ˂ 0.05). Other studies [22,30,31,32,34,35,83] using AC for the treatment of GP also reported significant therapeutic responses compared to the control groups (Table 1, Table 2, Table 3 and Table 4). 

Meng and Shi [25] observed an effect of herb-partitioned MX in patients with diabetic GP (Table 2). A total of 134 patients were randomised into a treatment and a control group. The treatment group was given herb-partitioned MX at *fanwei* point for 40 min, once a day, 5 times a week for six weeks, while the control group was given 50 mg of oral Itopride hydrochloride, 3 times a day. The therapeutic response was 92.5% in the treatment group (*p* < 0.05) compared to 74.6% in the control group. The study suggested that the effectiveness of herb-partitioned MX could be related to the regulation of plasma motilin and serum gastrin levels. Similarly, Guo et al. [26] studied 86 patients that were divided into two groups in which one group received MX and the other group received Banxia-Xiexin decoction combined with MX. Overall, the therapeutic rate was higher (97.7%) in the combination group compared to the MX-only group (88.4%). The authors concluded that MX combined with Banxia-Xiexin decoction could relieve the clinical symptoms of diabetic GP patients and lower the level of leptin with no obvious adverse reactions. 

The effect of HM in the patients suffering from gastroparesis symptoms was also noteworthy (Table 4). For example, Banxia-Xiexin decoction, Xiangshaliujunzi decoction, Chaishaoliujun decoction and hot packed Chinese medicine as HM were found to restore the gastric emptying rate and improve diabetic gastrointestinal symptoms in various studies included in this review. Liu et al. [52] studied the effect of concentrated Banxia-Xiexin on 76 diabetic gastroparesis patients in which the control group was treated with Domperidone for a period of 4 weeks. The results showed that Chinese herbal medicine was effective in lowering gastric emptying time. Similarly, Xiangshaliujunzi and hot packed Chinese medicine were efficient in improving gastric emptying rate and diabetic gastrointestinal symptoms [37,38,39,40,41,42,43,44,45,46,47,48]. 

Under the umbrella of HM, the efficacious impacts of Rumi Mastagi were investigated. Deriving from the *Pistacia lentiscus* plant, Rumi Mastagi was shown to improve diabetic GP concerning the GCSI significantly [78]. The study included 38 patients divided into a control and treatment group. Each group was treated with Rumi Mastagi and levosulpiride, respectively, for an 8-week window. The therapeutic response was a 20% acceleration for 4 h gastric emptying and a statistically significant reduction in mean gastric half-emptying time (T_1/2_) in both treatment and control groups.

### 3.2. Symptomatic Response

Both symptom scales and objective parameters which could be measured have been evaluated to determine the response to AC, EA, MX, and HM; these included gastroparesis cardinal symptom index (GCSI), gastric half emptying time (T_1/2_), 180 min—gastric residual rate (GRR), gastric drainage volume (GDV), STMP 16A (Serum transmembrane protein—ANO1), PM (Plasma motilin), PG (Plasma gastrin), SEG (Stomach electrogram), HAMA (Hamilton anxiety scale), HAMD (Hamilton depression scale), VCS (Various clinical symptoms), GP (Gastric peristalsis), GER (Gastric emptying rate), SG (Serum gastrin), motilin, PBGL (Postprandial blood glucose levels), PP (Pancreatic polypeptide), GGH (Glucose and glycated hemoglobin), FBG (Fasting blood glucose), nausea score, SS (Somatostatin), LP (Leptin), CER (Clinical effective rate), GIS (Gastrointestinal symptoms), QL (Quality of life), GH (Glycosylated hemoglobin), RAD (Reduction in abdominal distension, 2hPBG (2 h postprandial blood glucose). Almost all the studies reported some differences in the symptomatic outcomes before and after treatment. For example, Zhou et al. [18] reported increased statistical significance (*p* < 0.01) in the total GCSI score, T_1/2_, and 180 min—GRR in both treatment and control groups following treatment. GCSI score was also determined in several studies [19,20,21,25,29,36] and showed significant differences between treatment and control groups before and after treatment. Various clinical symptoms (VCS) were also addressed. For example, Zhang et al. [34] studied abdominal distension, belching, nausea-vomiting, upper-abdominal distention pain, sour regurgitation, and gastric burning sensation as VCS outcomes. The study stated that the clinical symptoms scores of both metoclopramide and control groups were decreased following treatment (*p* < 0.05). Comparable results were seen in other studies [28,35] in terms of VCS before and after treatment. Serum motilin level was used as an outcome by Wang et al. [19] in studies of the effectiveness of EA in diabetic patients with GP. However, the authors determined no significant changes in motilin levels between EA at Zusanli (ST 36) and Hegu (14) points compared to sham EA controls. In contrast, Guo et al. [26] found significant differences in motilin levels (*p* < 0.01) between treatment (340.82 ng/L) and control (422.42 ng/L) groups. Corresponding results were also obtained by Chang et al. [23] by applying electrical stimulation (2 Hz pulses for 30 min) on acupuncture points. Gastric drainage volume was another frequently reported outcome measure. For instance, in a study of 41 cases of post-surgical GP using MX, AC, and AC combined with auricular point sticking, Sun and Wang [17] stated that all three therapeutic procedures could decrease drainage volume. Two other studies [22,33] reported a significant reduction in GDV after treatment (*p* < 0.05).

## 4. Discussion

This systematic review explores the utility of alternative therapies for the management of gastroparesis. The therapies included AC, EA, MX, and HM. There are very few systematic reviews which have explored the treatment of GP with alternative therapies, and this review is our attempt to bridge that knowledge gap. AC is a frequently practiced, non-pharmacological treatment in the East Asian healthcare system which entails the placement of small needles into certain points in the body that are thought to be spots of ‘vital energy’ (the ‘qi’ of traditional Asian medicine) [84]. These points range from traditional acupuncture points and myofascial trigger points to nontraditional additional points and non-predefined new points, representing the practitioners’ different clinical expertise. The AC points that were targeted varied among the reported studies but most of the studies used CV 4, CV 6, CV 8, CV 10, CV 12, CV 13, ST 25, ST 36, SP 4, SP 9, SP 10, SP 6, SP 8, OV 12, BL 20, BL 21, BL 22, LI 11, LI 4, LR 14, ST 40, PC 6, MA-IC, MA-AH 7, MA-IC 3, MA-IC 4, AT 1, GV 20, EX-HN 3, PC 6, GB 34, and RN 12. AC has been shown to affect gastric motility through stimulating the vagus nerve [85], serotonergic networks [86], opioidergic processes [14], and spinal or supraspinal responses [85]. Mechanistically, acupuncture may support parasympathetic activity and reduce sympathetic output [14]. As such, this promotes pyloric relaxation, antral contractions and the modulation of enteric neurotransmitters acetylcholine and nitric oxide [87]. In this environment, the function of the ICC may be restored, and is demonstrated by increasing c-Kit expression, which bolsters ICC differentiation and prevents apoptosis [87]. Downstream regulation of gastrointestinal hormones such as motilin and ghrelin further supports slow-wave activity and gastric emptying [88]. Moreover, AC was found to influence gastric receptors in patients with gastroparesis [23], although this was not replicated in other studies [19]. Our review of the relevant studies [17,22,29,30,31,32,34,35,66,67,68,70,74,83] indicates that AC intervention was associated with higher therapeutic responses and improved clinical symptoms compared to control groups.

EA has been shown to alleviate or normalise stomach dysrhythmia and to enhance plasma pancreatic polypeptide levels via the vagus nerve [19,23]. The physiological mechanisms of EA mirror that of AC. However, with increasing vagal stimulation and release of acetylcholine, EA can function as therapy which can reliably increase gastric slow-wave coordination and peristalsis more reliably [89]. Furthermore, EA has been shown to regulate the expression of transient receptor prospective vanilloid 1 (TPRV1), a non-selective Ca^2+^-permeable cation channel with important physiological activities in the peripheral and central nervous systems [15,16]. Typically, EA or prokinetic medications are used to ease symptoms; however, patients’ responses to the therapy may vary. In the current review, EA treatment was associated with an improvement in clinical symptoms of patients after treatment [18,19,28]. The duration of the EA intervention varied, but the average duration was 12 days [18,19,28]. 

Moxibustion is an external treatment that consists of burning mugwort (*Artemisia vulgaris*) on specific spots on the body. It is based on the philosophy of traditional Chinese medicine (TCM). Moxibustion has been used to prevent and heal ailments for over 2500 years and can ‘dredge meridians’ and regulate qi-blood [90]. It reportedly can be utilised in as many as 364 different disorders according to a bibliometric review of studies published in China between 1954 and 2007. Meridians, cutaneous areas, and acupoints are all linked to MX. The meridian system is reportedly made up of channels and collaterals, which are conduits between internal and external systems, involving organs and the transmission of ‘qi-blood’, which purportedly regulates the entire body [90]. Distinctive acupoints may aid in the treatment of various diseases in MX including the treatment of GP. Specific to GP, emerging evidence suggests that MX mediates gastrointestinal regulatory mechanisms through thermal stimulation. Cutaneous sensory afferents activate TPRV1 channels, permitting Ca^2+^ influx to create action potentials that reach autonomic regulatory centres. When these signals have reached the nucleus tractus solitarius and subsequently, the dorsal motor nucleus of the vagus, vagal parasympathetic efferent signals are conducted to the stomach [15,16]. Yet, the therapeutic response to MX of patients with GP the studies reviewed here varied. Interestingly, all studies encompassing MX therapy showed significant results whether used as an individual treatment [17] or as combined with other therapies, such as herb-partitioned MX [25], Banxia-Xiexin decoction with MX [26], cupping with MX [27], or AC combined with MX [28]. 

Herbal medicines have historically been used for the treatment of gastrointestinal discomfort in many cultures. Banxia-Xiexin decoction (BXXD), a traditional Chinese herbal medicine comprising seven frequently utilised herbs (*Pinellia ternata*, *Radix scutellariae*, *Rhizoma zingiberis*, *Panax ginseng*, *Radix glycyrrhizae*, *Coptis chinensis*, and *Fructus jujubae*), has long been used in clinical practice in China to treat gastrointestinal discomfort. Similarly, Xiangshaliujunzi Decoction is a traditional Chinese medicine that contains eight common herbs (*Panax ginseng*, *Rhizoma atractylodis macrocephalae*, *Poria*, *Radix glycyrrhizae*, *Pericarpium citri reticulatae*, *Pinellia tuber*, *Fructus amomi*, *Radix aucklandiae*). Based on Traditional Chinese Medicine theory, its mechanism of action is thought to be linked to stimulating the spleen, regulating the stomach, facilitating elimination, and restoring the balance of Qi. Physiologically, several constituent herbs enhance gastric emptying and antral contractility through modulation of vagal pathways, increasing acetylcholine release [91]. Compounds from *Panax ginseng* and *Atractylodes macroephala* regulate ICC by increasing Ca^2+^-permeable cation channel activity to increase gastric peristalsis [91]. *Coptis chinensis* reduces pro-inflammatory cytokine activity, which contributes to the restoration of impaired gastric neuromuscular signalling in GP [92]. Additionally, these formulations may promote the release of motilin, ghrelin, and gastrin which promote gastric emptying and coordinated motility [93]. All studies reviewed here that pertained to intervention with HM reported positive results with respect to GER and CER [38,39,40,41,42,43,44,45,46,47,48,49,50,51,52,53,54,55,56,57,58,59,60,61,62,63,64,69,76,78,81].

In addition to improvements in measurable objective parameters of GP, there was also improvement in symptoms of gastroparesis patients who received AC, EA, MX, or HM treatments. The majority of studies indicated a significant improvement (*p* < 0.05) in Gastroparesis Cardinal Symptom Index (GSCI) score, which is widely used to evaluate symptoms of gastrointestinal disorders [19,20,21,25,29,36,65]. Similar positive findings were seen for GDV, although only three trials identified GDV as a clinical end-point [17,22,33]. A few studies measuring VCS levels noted that the untreated gastroparesis patient groups had increased VCS levels compared with the treated groups [28,34,35]. Most included trials failed to completely report side effects; all adverse events recorded were small, although we do not rule out the possibility of under-reporting. Only one study reported quality-of-life outcomes [47]. None of the studies reported changes in medication use in any of the included trials [94].

### Limitations

Similar to other systematic reviews, we encountered limitations in the literature related to assessing treatments for GP via AC, EA, MX, and HM. Most of the studies included in this review were from China, and it will be helpful to confirm the findings in other countries around the world. There might also be an element of publication bias. The randomization protocols or specifics of allocation concealment were not reported in the majority of RCTs. Furthermore, patient demographics were inadequately reported. The studies that were reviewed were diverse in design, methodological quality, specific interventions, and the characteristics of the study population; thus, limited availability of research design information may have contributed to potential study misclassification and skewed effect estimates. The majority of studies included in this review were of diabetic GP patients, and additional studies for post-surgical and idiopathic GP will be important. Further to this, no studies focussed on the efficacy of alternative therapies and GP in children. The outcome measures among the studies were not standardised. Further to this, there is a predominance of studies which focused on HM, which may have created a generalised bias to support the efficacy of all alternative therapies. Future studies should utilise objective measurements such as the Medical Outcomes Study (MOS) 36 Item Short Form Health Survey (SF-36), the functional digestive diseases quality of life questionnaire (FDDQL) [95], and the PAGI-QoL Upper Gastrointestinal Disorders-Quality of Life questionnaire [96].

## 5. Conclusions

GP is a debilitating disorder characterised by prolonged gastric emptying in the absence of mechanical gastric obstruction. Many effective therapies are available for the treatment of gastroparesis. This systematic review provides clinicians with information about alternative treatments, including AC, EA, MX, and HM for the management of GP. Based on the current evidence, we can conclude that AC, EA, MX, and HM can offer therapeutic alternatives for patients suffering from GP. However, we cannot yet recommend incorporation of these treatments into mainstream medicine due to the lack of well-designed and robust studies. Therefore, larger, well-designed RCTs including long-term follow-up will be required before AC, EA, MX, and HM can satisfactorily complement established therapies.

## Figures and Tables

**Table 1:** Characteristics of included studies with electroacupuncture.

Reference	Study	Patients (T/G)	Mode of Operation	Duration	Outcome	Comments
[18]	Zhou et al., 2020	63/2	ST 36, CV 12	Once a day, 5 times a week, 3 weeks	GCSI, T_1/2_, GRR,	NA
[19]	Wang et al., 2008	19/2	ST 36, LI 4	Four sessions over 2 weeks	GCSI, T_1/2_, SG, PM, PBGL, PP	Sham EA was taken as a control
[24]	Song et al., 2018	18	Transcutaneous electroacupuncture PC 6, ST 36	30 min for baseline, and 60 min for STEA	PP, NS	Real STEA vs. Sham STEA. STEA was effective in gastric dysrhythmia
[73]	Lin et al., 2024	48/2	CV12, ST25, LU1, LR13, SP3, LU9, ST36, ST37	Once daily, 30 min, 5 times a week for 2 weeks	GCSI, GSRS	Sham EA was provided to control. EA was effective in gastroparesis management
[75]	Pan et al., 2017	56/2	NA	Once daily, 30 min, 5 times a week for 2 weeks	GCSI, GER, GIS	No control, groups divided by mild and severe gastroparesis
[77]	Sarosiek et al., 2013	12	Transcutaneous electroacupuncture PC6, ST36	2 h, three times per day, over 4 weeks	GIS	All patients had both treatment and sham at different time points. EA was shown to be effective
[80]	Yue et al., 2023	51/2	CV12, ST36	Once daily, 30 min, 5 times a week for 3 weeks	T_1/2_, GRR	No control group, two groups based on multiple or single acupoints. Multiple considered more effective

T/G (Total number of patients/Number of treatment groups), GCSI (Gastroparesis cardinal scoring index), GRR (Gastric residual rate), T_1/2_ (Gastric half-emptying time), SG (Serum gastrin), STEA (Synchronised transcutaneous electroacupuncture), NS (Nauseas score) PBGL (Postprandial blood glucose levels), PP (Pancreatic polypeptide), GSRS (Gastroparesis symptom rating scale), PM (Plasma motilin), GER (Gastric emptying rate), GIS (Gastrointestinal symptoms), NA (Not applicable).

**Table 2:** Characteristics of included studies with moxibustion.

Reference	Study	Patients (T/G)	Intervention Combinations	Mode of Operation	Duration	Outcome	Comments
[17]	Sun and Wang, 2007	41/3	Acupuncture, moxibustion	NA	NA	GDV	Moxibustion considered the best method
[25]	Meng and Shi, 2020	134/2	Herb-partitioned moxibustion	Fanwei point	Once a day, 5 times a week, for 6 weeks	GCSI, SG, PM, TCM	Itopride hydrochloride medication for control
[26]	Guo et al., 2019	86/2	Moxibustion with Banxia-Xiexin decoction	NA	Four weeks	PM, SS, LP	Control group was treated with moxibustion
[27]	Hou and Yu et al., 2013	36/3	Moxibustion, Acupuncture and Cupping	BL 21, CV 13, CV 12, CV 10, ST 25, CV 8, ST 36	Once a day, 5 times during a course, with total 2 courses	GCSI	Moxibustion combined with cupping was efficient
[28]	Zhihai et al., 2014	100/4	Moxibustion, acupuncture, electroacupuncture, hydro acupuncture,	NA	3 courses	VCS	Acupuncture combined with moxibustion was effective among treatments
[71]	Li and Wang, 2024	48/2	Moxibustion, acupoint injection of methylcobalamin	ST36	Once daily, 4-week duration	GCSI	Control group was treated with metoclopramide and gastroparesis diet
[79]	Yan and Zeng, 2023	102/2	Moxibustion, ironing therapy	On umbilicus	Once daily, 4-week duration	GCSI, GER	Moxibustion and ironing therapy was shown to be effective

T/G (Total number of patients/Number of treatment groups), GDV (Gastric drainage volume), GCSI (Gastroparesis cardinal scoring index), PM (Plasma motilin), VCS (Various clinical symptoms), GER (Gastric emptying rate), SG (Serum gastrin), LP (Leptin), SS (Somatostatin), NA (Not applicable).

**Table 3:** Characteristics of included studies with acupuncture.

Reference	Study	Patients (T/G)	Mode of Operation	Duration	Outcome	Comments
[20]	Miller et al., 2014	8/2	PC 6, ST 36, LR 14, CV 12	12 weeks medication and 8 weeks for acupuncture with 2–3-week gap	GCSI, GER, GGH	First patients received domperidone for 12 weeks, then acupuncture for 8 weeks
[21]	Li et al., 2015	21/2	NA	7 days for both with one month gap	GCSI, FBG, GGH	The two groups received real or sham acupuncture
[22]	Sun et al., 2010	63/2	RN 12, ST 36, PC 6, SP 6,	Once a day	GDV	Metoclopramide was given to control three times a day
[23]	Chang et al., 2001	15	NA	For one minute, with 30 min interval	PP, SG, PM	Acupuncture followed by electrical stimulation with 30 min interval
[29]	Song et al., 2020	128/2	CV 12, ST 36, SP 9, SP 10, SP 6, SP 8	Once a day, 6 times a week, 4 weeks	GCSI, STMP 16A (ANO1)	Mosapride citrate was given to control group
[30]	Chen et al., 2012	42/2	ST 36, OV 12, BL 21	Once a day a week, 4 weeks	PM, PG	Domperidone was given to control group
[31]	Zeng et al., 2006	80/4	NA	Once a day a week, 2 weeks	SEG	Grouping in terms of acupuncture intensity and hypoglycemic drug as control
[32]	Zeng and Chai, 2008	60/2	CV 12, ST 36, PC 6, SP 6	Once a day, for 2 weeks	NA	Domperidone was given to control
[33]	Zhang 2010	20/2	BL 20, BL 21, BL 22, PC 6, CV 12, CV 6, ST 25, MA-IC, MA-AH 7, MA-IC 3, MA-IC 4, MA-AT 1, GV 20, EX-HN 3	Once a day, 5 times a week, for 4 weeks	HAMD, HAMA, GDV	Significant in terms of HAMD and HAMA
[34]	Zhang et al., 2014	46/2	ST 36, BL 21	Once a day, for 2 weeks	VCS	Metoclopramide for both groups
[35]	Zhang et al., 2007	72/2	LI 11, LI 4, CV 12, ST 36, ST 40, SP 9, SP 6, SP 10, SP 8	Twice each day, 10 days with interval of 2 days	NA	Domperidone was given to control group
[36]	Xuefen et al., 2020	99/3	CV 12, ST 36, PC 6,	Once a day, for 5 days, with 2 days gap b/w three courses	GCSI, GER	No control group, three groups based on acupoints
[66]	Cao et al., 2024	53/2	NA	Once a day for 5 days over 4 weeks	GCSI, FBG, 2hPBG	Control group was treated with mosapride
[67]	Chen et al., 2023	84/2	GV20, GV24, CV12, ST36, LI4, LR3	Once a day 3 times a week for 4 weeks	GCSI, TCM, FBG, 2hPBG, GH	Acupuncture treatment is effective with domperidone
[68]	Cheng et al., 2022	80/2	NA	Once a day for 2 weeks	GCSI, GIS, RAD	Control group was treated with itopride
[70]	Kotstitska et al., 2017	34/2	NA	5 sessions of 40 min, over 1 week	GCSI, GER	Control group was treated with itopride daily
[72]	Li et al., 2023	65/2	NA	Once a day for 30 min, 3 days	GCSI, GER, GDV	No control group, two groups based on acupoints (body/ear). Auricular considered more effective
[74]	Liu et al., 2024	99/3	CV12, ST36	30 min per day, 5 days a week for 3 weeks	GCSI, FPG, 2hPBG	Groups determined by multiple, single or sham acupoints. Combinations more effective
[82]	Wang 2004	80/3	CV 12, ST 36, PC 6, SP 4, BL 20, BL 21, LI 11, GB 34, CV 6, CV 4, SP 6	Once a day, for 10 days, with 5 days interval b/w two courses	VCS, GP	Two control groups, one given domperidone while other nothing

T/G (Total number of patients/Number of treatment groups), GCSI (Gastroparesis cardinal scoring index), GDV (Gastric drainage volume), PM (Plasma motilin), PG (Plasma gastrin), SEG (Stomach electrogastrogram), HAMD (Hamilton depression scale), HAMA (Hamilton anxiety scale), VCS (Various clinical symptoms), GP (Gastric peristalsis), GER (Gastric emptying rate), SG (Serum gastrin), STMP 16 A—ANO1 (Serum transmembrane protein), PP (Pancreatic polypeptide), GGH (Glucose and glycated hemoglobin), FBG (Fasting blood glucose), TCM (Traditional Chinese medicine), FPG (Fasting plasma glucose), GH (Glycosylated hemoglobin), RAD (Reduction in abdominal distension), 2hPBG (2 h postprandial blood glucose), NA (Not applicable).

**Table 4:** Characteristics of included studies with herbal medicine.

Reference	Study	Patients (T/G)	Type of Herbal Medicine	Duration	Outcome	Comments
[37]	Cai, 2011	54/2	Modified xiangshaliujunzi decoction	4 weeks	CER, GER, GIS	Control group was treated with metoclopramide
[38]	Feng 2001	128/2	Modified xiangshaliujunzi decoction	4 weeks	CER, GIS	Cisapride and metoclopramide as control group
[39]	Gou et al., 2009	62/2	Modified xiangshaliujunzi decoction	8 weeks	CER, GIS	Control group was treated with domperidone
[40]	Lu 2009	94/2	Modified xiangshaliujunzi decoction	4 weeks	CER, GIS	Domperidone as a control group
[41]	Lu et al., 2011	142/2	Modified xiangshaliujunzi decoction	4 weeks	CER, GER, GIS	Mosapride as a control group
[42]	Meng 2012	62/2	Modified xiangshaliujunzi decoction	4 weeks	CER, GIS	Cisapride and metoclopramide as a control group
[43]	Wen 2012	87/2	Modified xiangshaliujunzi decoction	4 weeks	CER, GER, GIS	Control group was treated with cisapride
[44]	Dai 2003	70/2	Modified xiangshaliujunzi decoction	3 weeks	CER, GER, GIS	Control group was treated with domperidone
[45]	Ji 2009	56/2	Modified xiangshaliujunzi decoction	2 weeks	CER, GIS	Domperidone as a control group
[46]	Hou et al., 2010	112/2	Modified xiangshaliujunzi decoction	8 weeks	CER, GIS, GER	Domperidone as a control group
[47]	Yan et al., 2013	50/2	Hot application with packed Chinese medicine	4 weeks	CER, GER, QL	Cisapride as a control group
[48]	Liang et al., 2015	66/2	Hot application with packed Chinese medicine	4 weeks	GER, FPG, CER, GH	NA
[49]	X et al., 2006	83/2	Modified Banxia-Xiexin decoction	9 weeks	CER, GER	Domperidone as a control group
[50]	Gao 2011	67/2	Modified Banxia-Xiexin decoction	8 weeks	CER, GER	Domperidone as a control group
[51]	Li 2004	85/2	Modified Banxia-Xiexin decoction	4 weeks	CER, GIS	Domperidone as a control group
[52]	Liu et al., 2008	76/2	Concentrated Banxia-Xiexin decoction	4 weeks	CER, GER	Domperidone as a control group
[53]	Liu 2012	120/2	Concentrated Banxia-Xiexin decoction	4 weeks	CER, GER	Domperidone as a control group
[54]	Luo et al., 2008	92/2	Modified Banxia-Xiexin decoction	4 weeks	CER, GER, GIS,	Mosapride as a control group
[55]	Qiu et al., 2004	65/2	Modified Banxia-Xiexin decoction	4 weeks	CER, GER	Cisapride as a control group
[56]	Sun 2009	96/2	Concentrated Banxia-Xiexin decoction	2 weeks	CER, GER, GIS	Domperidone as a control group
[57]	Wang 2011	100/2	Modified Banxia-Xiexin decoction	2 weeks	CER, GER, GIS	Domperidone as a control group
[58]	Wang 2011	56/2	Modified Banxia-Xiexin decoction	4 weeks	CER, GER, GIS	Domperidone as a control group
[59]	Wang 2010	96/2	Modified Banxia-Xiexin decoction	4 weeks	CER, GER	Domperidone and roxithromycin as a control group
[60]	Yin 2012	110/2	Concentrated Banxia-Xiexin decoction	4 weeks	CER, GER	Domperidone as a control group
[61]	Zhou 2003	72/2	Modified Banxia-Xiexin decoction	4 weeks	CER, GER	Domperidone as a control group
[62]	Zhou 2005	86/2	Modified Banxia-Xiexin decoction	4 weeks	CER, GER, FBG	Cisapride as a control group
[63]	Zhu and Ji 2009	50/2	Modified Banxia-Xiexin decoction	4 weeks	CER, GER	Domperidone as a control group
[64]	Zou 2009	48/2	Modified Banxia-Xiexin decoction	4 weeks	CER, GER, FBG	Domperidone as a control group
[69]	Liang, 2022	60/2	Xiangshaliujunzi decoction	3 weeks	CER, GIS	Control group was treated with cisapride
[76]	Liu, 2021	98/2	Chaishaoliujun decoction	2 weeks	CER, GIS, GER	NA
[78]	Singh et al., 2024	38/2	Rumi Mastagi	Twice daily for 8 weeks	GCSI, GH, T_1/2_	Control group was treated with levosulpiride
[81]	Zhu and Mei, 2022	122/2	Banxia-Xiexin decoction	4 weeks	CER, GER, GIS	Decoction proven to be effective over control (domperidone)

T/G (Total number of patients/Number of treatment groups), GCSI (Gastroparesis cardinal scoring index), T_1/2_ (Gastric half-emptying time), GER (Gastric emptying rate), CER (Clinical effective rate), GIS (Gastrointestinal symptoms), QL (Quality of life), NA (Not applicable).

## Data Availability

No new data were created or analysed in this study. Data sharing is not applicable to this article.

## Abbreviations

The following abbreviations are used in this manuscript:

GP	Gastroparesis
AC	Acupuncture
EA	Electroacupuncture
MX	Moxibustion
HM	Herbal medicine
